# Multidisciplinary treatment in children with ectodermal dysplasia

**DOI:** 10.1186/1746-160X-8-S1-P5

**Published:** 2012-05-25

**Authors:** J Norderyd

**Affiliations:** 1 National Oral Disability Centre, Jönköping, Sweden

## 

Oral habilitation in children with ectodermal dysplasia (ED) necessitates consideration of the special dental and oral conditions as well as of the growing child’s physical and psychological development. The severity of ED symptoms varies and is for some families a source of daily worries and complications. The paediatric dentist may be the first professional to suggest the diagnosis of ED since delayed eruption of teeth, aberrant tooth shape and oligodontia are distinct symptoms. Oligodontia may affect chewing ability, speech and aesthetics, so early treatment is often favourable in order to give the child good oral function and appearance. Prevention of dental fear and anxiety is important, since the individual with ED is likely to need dental treatment repeatedly during childhood, adolescence and young adulthood. Therefore, behaviour management methods in combination with an adequate use of sedation and pain control measures are essential.

Due to the complexity of treatment planning for children with oligodontia, a multidisciplinary team approach is beneficial and the team should preferably include the disciplines of paediatric dentistry, orthodontics, prosthetic dentistry, and maxillofacial surgery. The patient and the parents should always be involved in the treatment planning and be informed about short- and long-term treatment options. Aesthetic improvement of existing teeth should be done early if requested. Removable dentures at an early age may also be beneficial for some children. Treatment with fixed dental prostheses should be evaluated at early school age according to individual needs and indications. Examples of multidisciplinary treatment in three children with ED treated in the framework of free dental care for children in Sweden will be presented.

